# Effects of sporadic inclusion body myositis on skeletal muscle fibre type specific morphology and markers of regeneration and inflammation

**DOI:** 10.1007/s00296-024-05567-8

**Published:** 2024-04-06

**Authors:** Kasper Yde Jensen, Jakob Lindberg Nielsen, Per Aagaard, Mikkel Jacobsen, Anders Nørkær Jørgensen, Rune Dueholm Bech, Ulrik Frandsen, Louise Pyndt Diederichsen, Henrik Daa Schrøder

**Affiliations:** 1 Copenhagen Research Center for Autoimmune Connective Tissue Diseases (COPEACT), Center for Rheumatology and Spine Diseases, Copenhagen University Hospital, Rigshospitalet, Juliane Maries Vej 10, 2100 Copenhagen, Denmark; 2 Department of Sports Science and Clinical Biomechanics, University of Southern Denmark, Odense, Denmark; 3 Department of Pathology, Department of Clinical Research, University of Southern Denmark, Odense University Hospital, Odense, Denmark; 4 Department of Clinical Research, University of Southern Denmark, Odense, Denmark; 5 Department of Orthopaedics and Traumatology, Zealand University Hospital, Koege, Denmark; 6 Department of Rheumatology, Odense University Hospital, Odense, Denmark

**Keywords:** Myopathies, Immunology, Satellite cells, Macrophages, Myonuclei

## Abstract

Sporadic inclusion body myositis (sIBM) is a subgroup of idiopathic inflammatory myopathies characterised by progressive muscle weakness and skeletal muscle inflammation. Quantitative data on the myofibre morphology in sIBM remains scarce. Further, no previous study has examined fibre type association of satellite cells (SC), myonuclei number, macrophages, capillaries, and myonuclear domain (MD) in sIBM patients. Muscle biopsies from sIBM patients (*n* = 18) obtained previously (NCT02317094) were included in the analysis for fibre type-specific myofibre cross-sectional area (mCSA), SCs, myonuclei and macrophages, myonuclear domain, and capillarisation. mCSA (*p* < 0.001), peripheral myonuclei (*p* < 0.001) and MD (*p* = 0.005) were higher in association with type 1 (slow-twitch) than type 2 (fast-twitch) fibres. Conversely, quiescent SCs (*p* < 0.001), centrally placed myonuclei (*p* = 0.03), M1 macrophages (*p* < 0.002), M2 macrophages (*p* = 0.013) and capillaries (*p* < 0.001) were higher at type 2 fibres compared to type 1 fibres. In contrast, proliferating (Pax7^+^/Ki67^+^) SCs (*p* = 0.68) were similarly associated with each fibre type. Type 2 myofibres of late-phase sIBM patients showed marked signs of muscle atrophy (i.e. reduced mCSA) accompanied by higher numbers of associated quiescent SCs, centrally placed myonuclei, macrophages and capillaries compared to type 1 fibres. In contrast, type 1 fibres were suffering from pathological enlargement with larger MDs as well as fewer nuclei and capillaries per area when compared with type 2 fibres. More research is needed to examine to which extent different therapeutic interventions including targeted exercise might alleviate these fibre type-specific characteristics and countermeasure their consequences in impaired functional performance.

**Supplementary Information:**

The online version contains supplementary material available at 10.1007/s00296-024-05567-8.

## Introduction

Sporadic inclusion body myositis (sIBM) is an acquired slowly progressing disorder within the group of idiopathic inflammatory myopathies [1, 2]. Severe progressive muscle weakness accompanied by impairments in physical function are typical clinical traits of the disease [3, 4]. Histologically, sIBM is characterised by muscle fibre damage, inflammatory infiltrates, and over time, marked myofibre atrophy [2, 5, 6].

Studies investigating pathophysiological changes in sIBM muscles have mainly focused on the immune response, including lymphocytes [7, 8], macrophages [9, 10] and major histocompatibility complex [11], while studies describing other histological features such as myofibre size (cross-sectional area: mCSA), myofibre type distribution and the content of myogenic precursor cells are scarce [12–14]. Specifically, no previous studies have examined myofibre type-specific (type 1, slow-twitch and type 2, fast-twitch) association of satellite cells (SC), myonuclei, myofibre area per myonuclei (i.e., myonuclear domain: MD) and capillary supply in sIBM skeletal muscle.

Therefore, by analysing fibre-specific myogenic regenerative capacity (SCs and myonuclei), markers of inflammation (M1- and M2 macrophages), and vascularization the present study aimed to expand our understanding of the pathophysiological changes in myocellular morphology related to sIBM.

## Methods

### Patient characteristics

General characteristics of the included sIBM patients (*n* = 18) are presented in Table 1. The average age was 68.8 ± 5.9 years and the age at onset of symptoms was 60.1 ± 6.9 years. The patient group included 14 males (78%) and 4 females (22%). Seven patients (39%) were receiving immunosuppressive medication.

**Table 1:** Baseline characteristics of included patients

	sIBM patients (*n* = 18)
Age, (years)	68.8 ± 5.9
Male, (*n* (%))	14 (78%)
BMI, (kg/m^2^)	25.6 ± 4.2
Age first symptoms, (years)	60.1 ± 6.9
Time since first symptoms, (months)	104.7 ± 71.5
Creatine kinase (U/L) Reference value, (40–280)	449 ± 380
Health Assessment Questionnaire, (0–3)	0.98 ± 0.79
MMT8, (0–80)	68.8 ± 5.8
Immunosuppressive medicine, (*n* (%))	7 (39%)
Muscle biopsy M. Tibialis anterior, (*n* (%)) M. Vastus lateralis, (*n* (%))	14 (78%) 4 (22%)

Data are presented as mean ± SD or number & (%)

*BMI* Body Mass Index, *MMT8* Manual muscle testing 8, *MMT8* Manual muscle testing of 8 muscles

### Study outline

The muscle biopsy material from a previous randomised controlled trial (RCT) conducted in our lab (NCT02317094) was analysed [15]. Only baseline biopsies were included in the present study, comprising muscle biopsies from 21 biopsy-validated sIBM patients. Three biopsies were excluded, as the quantitative analysis was impossible due to excessive tissue damage (Figure A, Supplementary Material) and thus the data from 18 sIBMs muscle biopsies were included in the final analysis. Recruitment procedures have been described in detail previously [15].

### Muscle biopsy sampling

Muscle biopsies from m. vastus lateralis (VL) or in cases of severe VL atrophy, m. tibialis anterior (TA) were obtained using the Bergström needle (4.0 mm) technique. Immediately after extraction, the muscle samples were embedded in Tissue Tek (4583, Sakura Finetek, Alphen aan den Rijn, The Netherlands) and frozen in nitrogen-cooled 2-methyl butane. The muscle biopsies were stored in a freezer at − 80 °C until further analysis.

### Immunohistochemistry and data processing

Immunohistochemistry procedures were performed as described in detail elsewhere [10]; see Supplementary Table A for a detailed listing of the antibodies used.

The data processing with Visiopharm has been described previously [10]. In brief, the image analysis software (Visiopharm A/S, Denmark) was used following a three-step protocol: (1) Identifying muscle fibres. (2) Identifying and quantification of structures of interest (i.e., satellite cells). The linear distance from any fibre determined the allocation of structures of interest, in terms of fibre type (i.e., closest fibre). (3) Normalising the number of structures of interest to the analysed area (counts/mm^2^). For type 1 and type 2 fibres, the average analysed area was 1.94 ± 0.19 and 0.74 ± 0.01 mm^2^ per biopsy sample, respectively. To allow comparisons to the existing literature both density (cells/mm^2^) and structure of interest/fibre profile” for SCs, macrophages and capillaries are presented in the results section (Tables 3, 4, 5).

Analysis of mCSA, sublaminal myonuclei and centrally-placed myonuclei was conducted by manual analysis (AxioVision SE64 Rel. 4.9.1 software: Carl Zeiss, Germany). First, all cross-sectional fibres were identified, hereafter the cross-sectional area and the relative number of type 1 and 2 fibres, as well as myonuclei and central myonuclei, were recorded. A median of 120 fibres (range [21–837]) were analysed per biopsy (type 1; 71 [10–295] fibres, type 2; 62 [11–542]).

### Statistical analysis

All data reported in the present study were analysed for normality and homogenous distribution of variance, which could not be verified uniformly for all outcome parameters. The Wilcoxon’s signed-rank test was used to evaluate differences in outcome parameters between type 1 and 2 myofibres. All descriptive data are presented as group mean ± SD unless otherwise stated. The statistical significance level was set at *p* ≤ 0.05 (two-tailed testing). Statistical analysis was performed using RStudio (Version 2022.7.1.554) [16].

## Results

### Myofibre cross-sectional area, myonuclei and myonuclear domain

**Fig. 1:**
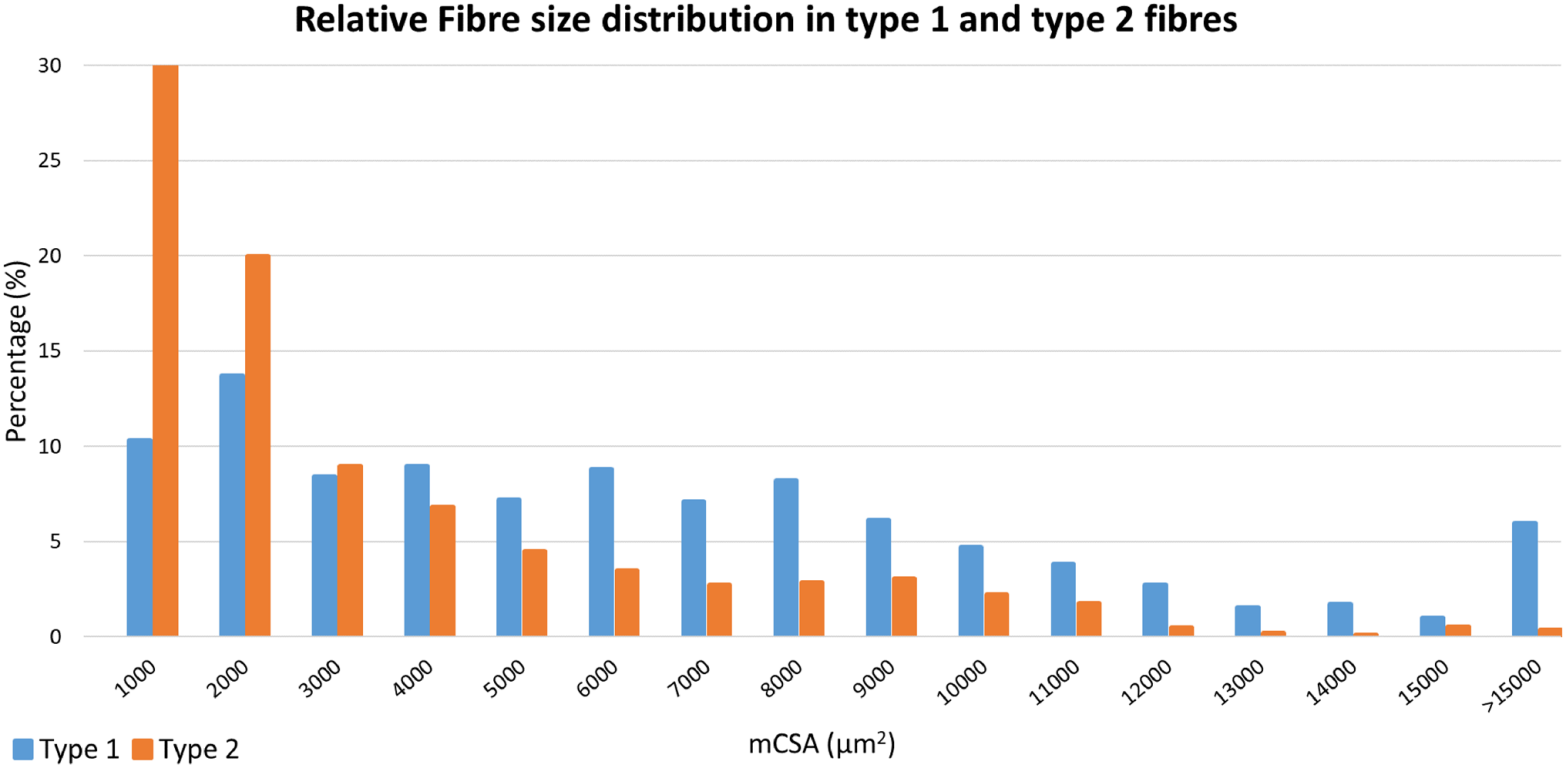
Relative fibre size distribution in type 1 and type fibres. Blue bars represent type 1 fibres. Orange bars represent type 2

Mean type 1 fibre mCSA (6040 ± 3054 µm^2^) was ~ 140% larger than type 2 fibre mCSA (2518 ± 1815 µm^2^) (*p* = 0.0002). Histograms depicting fibre type-specific mCSA distribution are shown in Fig. 1.

**Fig. 2:**
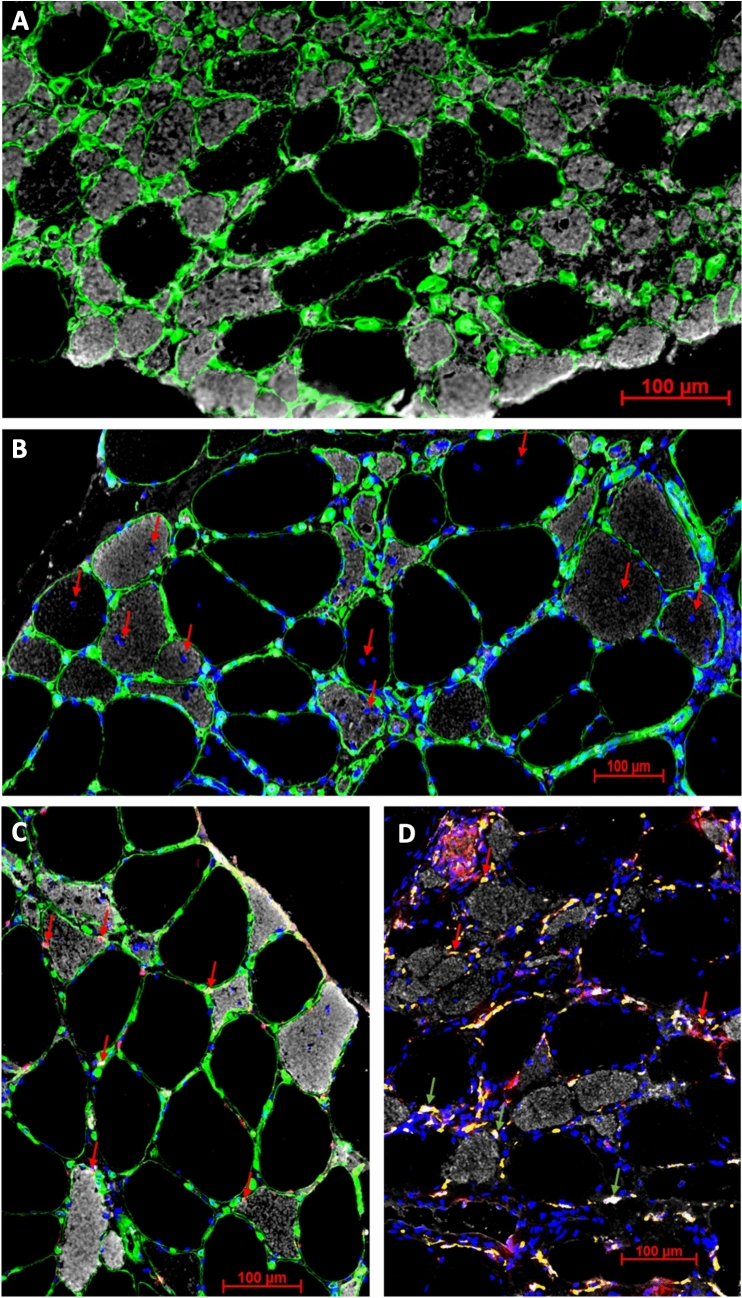
Illustrations of muscle biopsy findings from patients with sporadic inclusion body myositis (sIBM) **A** Type 1 fibres (black) were observed to be larger in size than type 2 fibres (grey). **B** The number of centrally placed myonuclei (red arrows) was higher in type 2 than in type 1 fibres. **C** The number of satellite cells (red arrows) was higher in association to type 2 fibres than to type 1 fibres. **D** M1 (red arrows) and M2 (green arrows) macrophages were observed to group into clusters in the vicinity of small type 2 fibres. **A** Green = laminin, Grey = MHC fast. **B** Green = laminin, Grey = MHC fast, blue = DAPI. **C** Green = laminin, Grey = MHC fast, blue = DAPI, red = pax7. Grey = MHC fast, blue = DAPI, yellow = CD206, red = CD68

Type 1 fibres demonstrated ~ 61% higher myonuclei (Six1^+^/DAPI) content per fibre profile as compared to type 2 fibres (*p* < 0.0001). Despite that myonuclear domain was ~ 65% larger in type 1 fibres as compared to type 2 fibres (*p* = 0.005) (Table 2). The number of centrally placed myonuclei (Six1^+^/DAPI) was 67% higher in type 2 than in type 1 fibres (*p* = 0.03) (Table 2); see Fig. 2 for examples of fibre type differences in size (A) and centrally placed myonuclei (B).

**Table 2:** Myofibre cross-sectional areas (mCSA) and myonuclei

	Time since first symptom (Months)	Muscle	mCSA Type 1 (µm^2^)	mCSA Type 2 (µm^2^)	Myonuclei (Six1^+^/DAPI) Type 1, (nuclei/fibre profile)	Myonuclei (Six1^+^/DAPI) Type 2, (nuclei/fibre profile)	Central myonuclei (Six1^+^/DAPI^+^) Type 1, (nuclei/fibre profile)	Central myonuclei (Six1^+^/DAPI^+^) Type 2, (nuclei/fibre profile)	Myonuclear domain Type 1 (µm^2^/nuclei)	Myonuclear domain Type 2 (µm^2^/nuclei)
Patient 1	100	Tibialis anterior	7479	4750	2.95	2.71	0.20	1.29	2720	1461
Patient 2	52	Tibialis anterior	4836	1342	3.00	1.47	0.21	0.31	1574	944
Patient 3	108	Tibialis anterior	7953	2160	3.55	1.98	0.16	0.40	2267	1171
Patient 4	173	Tibialis anterior	4270	2577	2.58	2.15	0.52	0.59	1696	1166
Patient 5	37	Tibialis anterior	6027	6778	2.67	3.00	0.012	0.17	2415	2474
Patient 6	86	Tibialis anterior	8354	1197	3.52	1.37	0.43	0.55	2300	765
Patient 7	208	Tibialis anterior	7145	923	2.85	1.08	0.074	0.58	2627	847
Patient 8	100	Tibialis anterior	1269	489	1.88	0.90	0.67	0.31	718	451
Patient 9	314	Vastus lateralis	2258	2826	1.72	1.84	0.19	0.32	1539	1727
Patient 10	101	Vastus lateralis	6638	6032	2.75	2.43	0.65	0.34	2608	2702
Patient 11	101	Tibialis anterior	12,918	2596	4.69	1.86	0.094	0.58	2554	1208
Patient 12	71	Vastus lateralis	2941	2285	2.45	2.13	0.30	0.20	1190	1174
Patient 13	143	Tibialis anterior	4325	2314	2.62	2.88	0.20	0.50	1717	772
Patient 14	66	Tibialis anterior	8639	4200	3.01	1.89	0.16	0.36	3163	1749
Patient 15	60	Tibialis anterior	6597	1374	3.01	1.57	0.27	0.35	2033	693
Patient 16	120	Tibialis anterior	1819	942	2.00	1.27	0.20	0.45	772	835
Patient 17	17	Vastus lateralis	4937	704	2.83	1.13	0.20	0.19	1765	510
Patient 18	28	Tibialis anterior	10,315	1840	4.52	1.74	0.23	0.65	2053	758
Mean	104.7		6040	2518*	2.91	1.81*	0.27	0.45*	1984	1189*
SD	71.5		3054	1815	0.78	0.62	0.19	0.25	673	628
*p* value			0.0002		< 0.001		0.03		0.005	

*Significantly different from type 1

### Satellite cells

Quiescent SC number associated with type 2 fibre area was higher as compared to that of type 1 fibre area (*p* < 0.0001), and this difference that persisted in the fibre profile analysis (i.e., more SCs/myofibre profile) (Table 3); see Fig. 2C for an example of fibre type differences in quiescent SCs.

**Table 3:** Satellite cells – cells/mm^2^ vs cells/fibre profile

	Quiescent SCs (Pax7^+^/Six1^+^) Type 1, (cells/mm^2^)	Quiescent SCs (Pax7^+^/Six1^+^) Type 2, (cells/mm^2^)	Quiescent SCs (Pax7^+^/Six1^+^) Type 1, (cells/fibre profile)	Quiescent SCs (Pax7^+^/Six1^+^) Type 2, (cells/fibre profile)	Proliferating SCs (Pax7^+^/ki67^+^) Type 1, (cells/mm^2^)	Proliferating SCs (Pax7^+^/ki67^+^) Type 2, (cells/mm^2^)	Proliferating SCs (Pax7^+^/ki67^+^) Type 1, (cells/fibre profile)	Proliferating SCs (Pax7^+^/ki67^+^) Type 2, (cells/fibre profile)
Patient 1	32.9	82.0	0.25	0.39	0.9	20.1	0.007	0.1
Patient 2	69.3	231.1	0.34	0.31	14.6	14.2	0.07	0.02
Patient 3	31.0	124.4	0.25	0.27	1.3	3.9	0.01	0.008
Patient 4	86.0	274.2	0.37	0.71	2.1	0	0.009	0
Patient 5	19.4	102.0	0.12	0.69	16.0	4.8	0.1	0.03
Patient 6	47.4	478.5	0.40	0.57	0.6	0	0.005	0
Patient 7	44.9	232.2	0.32	0.21	4.4	11.8	0.03	0.01
Patient 8	125.8	445.1	0.16	0.22	0	0	0	0
Patient 9	8.9	11.0	0.02	0.03	4.7	0	0.01	0
Patient 10	18.9	27.6	0.13	0.17	0	0	0	0
Patient 11	32.1	88.5	0.41	0.23	11.7	23.8	0.2	0.06
Patient 12	23.2	176.2	0.07	0.40	14.8	8.0	0.04	0.02
Patient 13	29.0	217.3	0.13	0.50	5.4	6.7	0.02	0.02
Patient 14	30.4	89.0	0.26	0.37	0.5	1.1	0.004	0.005
Patient 15	54.6	324.7	0.36	0.45	0.5	0	0.003	0
Patient 16	19.5	252.6	0.04	0.24	10.5	0	0.02	0
Patient 17	41.0	200.3	0.20	0.14	12.9	19.5	0.06	0.01
Patient 18	28.7	255.9	0.30	0.47	2.3	2.1	0.02	0.004
Mean	41.3	200.7*	0.23	0.35*	5.7	6.5	0.03	0.02
SD	28.4	129.9	0.13	0.19	5.9	8.0	0.04	0.03
P values	< 0.0001		0.01		0.68		0.08	

*Significantly different from type 1

Proliferating SCs was equally associated with type 1 fibre and type 2 fibre in both the area analysis and the fibre profile analysis (*p* = 0.08) also when compared with area (*p* = 0.68; *p* = 0.08, respectively) (Table 3).

### Macrophages

M1 macrophage density in type 2 fibre area was higher as compared to that of type 1 fibre area (*p* = 0.002). Likewise, M2 macrophages were more densely packed around type 2 fibres (*p* = 0.01) (Table 4).

**Table 4:** Macrophages – cells/mm^2^ vs cells/fibre profile

	M1 macrophages (CD68^+^/CD206^−^) Type 1, (cells/mm^2^)	M1 macrophages (CD68^+^/CD206^−^) Type 2, (cells/mm^2^)	M1 macrophages (CD68^+^/CD206^−^) Type 1, (cells/fibre profile)	M1 macrophages (CD68^+^/CD206^−^) Type 2, (cells/fibre profile)	M2 macrophages (CD68^+^/CD206^+^) Type 1, (cells/mm^2^)	M2 macrophages (CD68^+^/CD206^+^) Type 2, (cells/mm^2^)	M2 macrophages (CD68^+^/CD206^+^) Type 1, (cells/fibre profile)	M2 macrophages (CD68^+^/CD206^+^) Type 2, (cells/fibre profile)
Patient 1	12.9	40.6	0.10	0.19	6.9	6.8	0.05	0.03
Patient 2	95.8	241.6	0.46	0.32	162.0	211.4	0.78	0.28
Patient 3	47.5	144.0	0.38	0.31	41.0	132.2	0.33	0.29
Patient 4	94.5	57.0	0.40	0.15	25.2	34.20	0.11	0.09
Patient 5	19.7	32.7	0.12	0.22	7.3	20.4	0.04	0.14
Patient 6	62.7	163.7	0.52	0.20	28.0	229.2	0.23	0.27
Patient 7	38.2	104.8	0.27	0.10	31.3	104.8	0.22	0.10
Patient 8	77.7	146.8	0.10	0.07	0	16.3	0	0.01
Patient 9	9.2	9.3	0.02	0.03	6.9	5.2	0.02	0.01
Patient 10	13.1	12.8	0.09	0.08	2.4	2.8	0.02	0.02
Patient 11	63.8	228.2	0.82	0.59	83.4	190.1	1.08	0.49
Patient 12	0	0	0	0	11.3	16.0	0.03	0.04
Patient 13	32.4	169.0	0.14	0.39	10.2	84.5	0.04	0.20
Patient 14	22.4	52.4	0.19	0.22	5.8	31.8	0.05	0.13
Patient 15	21.6	66.7	0.14	0.09	6.5	18.6	0.04	0.03
Patient 16	0	0	0	0	0	0	0	0
Patient 17	60.6	353.1	0.30	0.25	116.3	564.4	0.57	0.40
Patient 18	36.8	189.4	0.38	0.35	26.6	289.8	0.27	0.53
Mean	39.4	111.8*	0.25	0.20	31.7	108.8*	0.22	0.17
SD	30.5	99.4	0.22	0.16	44.6	145.9	0.30	0.17
*p* values	**0.002**		0.15		**0.01**		**0.34**	

*Significantly different from type 1

In contrast, in the individual fibre profile analysis, both M1 & M2 macrophage number associated with type 1 were observed to be similar compared to type 2 fibres (*p* = 0.15; *p* = 0.34, respectively) (Table 4); see Fig. 2D for an example of fibre type differences in M1 and M2 macrophages.

### Capillarisation

Capillary density was of a higher abundance in type 2 fibre area compared to type 1 fibre area (*p* = 0.0002) (Table 5). However, if related to the fibre profile, type 1 fibres had more capillaries associated with the individual fibre as compared to type 2 fibres (*p* = 0.008) (Table 5).

**Table 5:** Capillaries – cells/ mm^2^ vs cells/fibre profile

	Capillary density (CD31^+^) Type 1, (cells/mm^2^)	Capillary density (CD31^+^) Type 2, (cells/mm^2^)	Capillary density (CD31^+^) Type 1, (cells/fibre)	Capillary density (CD31^+^) Type 2, (cells/fibre)
Patient 1	270	405	2.02	1.92
Patient 2	338	745	1.63	1.0
Patient 3	324	711	2.58	1.54
Patient 4	454	571	1.94	1.47
Patient 5	267	400	1.61	2.71
Patient 6	304	592	2.54	0.71
Patient 7	320	1034	2.29	0.95
Patient 8	361	540	0.46	0.26
Patient 9	161	101	0.36	0.29
Patient 10	179	170	1.19	1.03
Patient 11	299	554	3.86	1.44
Patient 12	266	437	0.78	1.0
Patient 13	347	555	1.50	1.28
Patient 14	197	219	1.70	0.92
Patient 15	291	392	1.92	0.54
Patient 16	441	751	0,80	0.71
Patient 17	474	831	2.34	0.59
Patient 18	529	587	5.46	1.08
Mean	323	533*	1.94	1.08*
SD	101	237	1.22	0.60
*p* values	< 0.001		0.008	

*Significantly different from type 1

## Discussion

As evaluated for the first time in myositis patients, we observed that mCSA, peripheral myonuclei, myonuclear domain was higher in association to type 1 (slow-twitch) as compared to type 2 (fast-twitch) myofibres. Conversely, quiescent SCs and central myonuclei, were higher in association to type 2 fibres compared to type 1 fibres. For both M1 and M2, the density was observed to be higher in type 2 fibres, however no differences were observed on the “per fibre profile” analyses. No fibre type-specific differences were observed for proliferating SCs. These observations suggest distinct and exclusive pathological changes related to the respective fibre types.

### Type 1 hypertrophy and type 2 atrophy

In the present study, mCSA was ~ 140% larger in type 1 fibres as compared to type 2 fibres, which aligns with previous biopsy data (70–100% larger) obtained in sIBM patients [12, 13]. In comparison, healthy age-matched adults (~ 65 years of age) typically demonstrate type 1 fibres that are between 10% smaller and up to ~ 15% larger than type 2 fibres [17–20]. Our mCSA histogram analysis identified a high number of pathologically large myofibres, sometimes termed *megafibres *[21], that contributed to the increased mean mCSA, especially for type 1 fibres (cf. Figure 1). Karlsen et al. reported healthy age-matched muscle (VL) to contain less than 10% of type 1 fibres with an mCSA above 7000 µm^2^ [22], while in the present cohort of sIBM patients ~ 40% of type 1 fibres demonstrated mCSA > 7000 µm^2^ (cf. Fig. 1).

In healthy lower limb (VL) muscles of adults between 50 and 70 years of age, mCSA of type 2 fibres typically amounts to 4000–6000 µm^2^, depending on habitual physical activity levels [23]. Specifically, in age-matched muscle biopsies obtained at ~ 65 years of age only a few (< 10%) type 2 fibres demonstrate mCSA ≤ 2000 µm^2^, irrespective of training history [22]. In contrast, the present group of sIBM patients showed highly marked myofibre atrophy, as 62% of type 2 fibres demonstrated mCSA ≤ 2000  µm^2^ (cf. Fig. 1 and Suppl. Figure B). These findings underline that the sIBM disease process not only severely affects lower limb muscle fibre morphology in accordance with the previous reports [12, 13], but also supports the findings in a recently published study by Nelke et al., that to our knowledge, is the first to reveal that the atrophic effect of the disease appears to be highly fibre type-specific, preferentially affecting fast-contracting type 2 myofibres [24].

### Type 2 fibre atrophy and higher association of quiescent satellite cells

Quiescent SC (Pax7^+^/Six1^+^) density associated with type 2 fibres was 52% higher compared to type 1 (cf. Table 3), even though type 1 fibres were larger. In contrast, the number of proliferating SCs (Pax7^+^/Ki67^+^) was similarly between type 1 and 2 fibres.

Overall myonuclei number (Six1^+^/DAPI) was ~ 61% higher in type 1 than type 2 fibres (cf. Table 3), which likely was driven by the larger mCSA of type 1 fibres, given that myonuclei number is known to be positively correlated with fibre size [22, 25, 26]. In contrast, the number of centrally placed myonuclei (Six1^+^) was ~ 67% higher in type 2 fibres (cf. Table 3), an observation that may be linked to the apparent more substantial type 2 myofiber atrophy.

The present cohort of sIBM patients demonstrated an enlarged (+ 67%) myonuclear domain in type 1 fibres compared to type 2 fibres. This could indicate that the proliferative capacity of satellite cells and their ability to fuse with the type 1 myofibers to donate their myonuclei may be dysregulated, as the mCSA to myonuclei ratio (i.e., myonuclei domain) have been demonstrated to be highly positively associated [27, 28].

### Higher degree of inflammation associated with type 2 fibres

Both M1 (CD68 + /CD206-) and M2 (CD68 + /CD206 +) macrophages were found to be threefold more abundant related to type 2 fibres as compared to type 1 fibres. These observations indicate higher levels of proinflammatory activity (M1), and elevated tissue remodelling (M2) associated to type 2 vs. type 1 fibres in sIBM patients. The present study did not obtain fibre type specific data on T-cell content. However, we previously observed spatial overlap between macrophage infiltration and T-cell accumulation in the present sIBM patients, where macrophage infiltration was dominantly concentrated around groups of highly atrophied type 2 fibres [10]. Collectively, the previous and present observations suggest that a fibre type dependent pattern of “disease activity” may exist with sIBM, characterised by vastly atrophied type 2 myofibres, which could be driven by the defective muscle regeneration seen in sIBM and myositis [29, 30].

### Metabolically suffering type 1 fibres

The number of capillaries (CD31^+^) density was greater (~ 65%) in relation to type 2 fibre area than type 1 fibre area. In contrast, observations in healthy age-matched adults typically demonstrate higher capillarisation in type 1 fibres compared to type 2 fibres [20, 31]. This disparity suggests that the sIBM disease might be affecting capillary content, however, based on the present observations it is difficult to conclude whether the disease induces increased type 2 fibre capillarisation, or decreased type 1 fibre capillarisation or both. The lower abundance of capillaries of type 1 fibre area, taken together with the higher myonuclear domain of type 1 fibres (lower abundance of myonuclei per fibre profile), could suggest that type 1 fibres in sIBM are metabolically “suffering”, due to negligence in repair and blood supply. This suggests that the sIBM disease might be affecting angiogenesis, especially within type 1 fibres.

### Study limitations

The present data were obtained in a somewhat small and heterogeneous group of sIBM patients, which were characterized by large inter-individual differences in disease duration, plasma creatine kinase levels and immunosuppressive treatment. As the sample size did not allow for more detailed subgroup analysis, cautions should be made against generalisations based on the current findings.” The majority of the muscle biopsies were acquired from m. tibialis anterior, rather than m. vastus lateralis, which could have affected the data, however previous data show no significant difference in inflammation or pathologic features between these muscles [32, 33]. Owing to a high degree of physical inactivity and very pronounced muscle atrophy in some study participants, a number of muscle samples did not allow for quantification of mCSA, SC number, myonuclei content etc. and therefore could not be included in the overall statistical analysis (cf. suppl. Figure A), thus reducing the sample size.

## Conclusions

As reported for the first time, sIBM appears to exert differential effects on the morphology as well as density and content of quiescent satellite cells, myonuclei, M1 and M2 macrophages and capillarisation of type 1 and type 2 myofibres. In contrast, no fibre type differences in number of associated proliferating satellite cell could be observed. Somewhat paradoxically, type 2 muscle fibres were characterized by focally elevated levels of quiescent satellite cells and central myonuclei, yet these fibres remained markedly atrophied. Likewise, type 1 fibre morphology was pathologically comprised with the presence of megafibres accompanied by large myonuclear domains. All suggest an impact of the sIBM disease on fibre morphology, presumably due to a preferential imbalance between myofibrillar regeneration and degeneration.

### Supplementary Information

Below is the link to the electronic supplementary material.

Supplementary file1 (TIF 9654 KB)

Supplementary file2 (TIF 957 KB)

Supplementary file3 (DOCX 15 KB)

## Data Availability

Data are generally not available due to current GDPR regulations.
