# Supplementary figures and images for: Effects of sporadic inclusion body myositis on skeletal muscle fibre type specific morphology and markers of regeneration and inflammation
Source: Rheumatol Int. 2024 Apr 6;44(6):1077–87. doi: 10.1007/s00296-024-05567-8 (PMC11108868; doi:10.1007/s00296-024-05567-8)

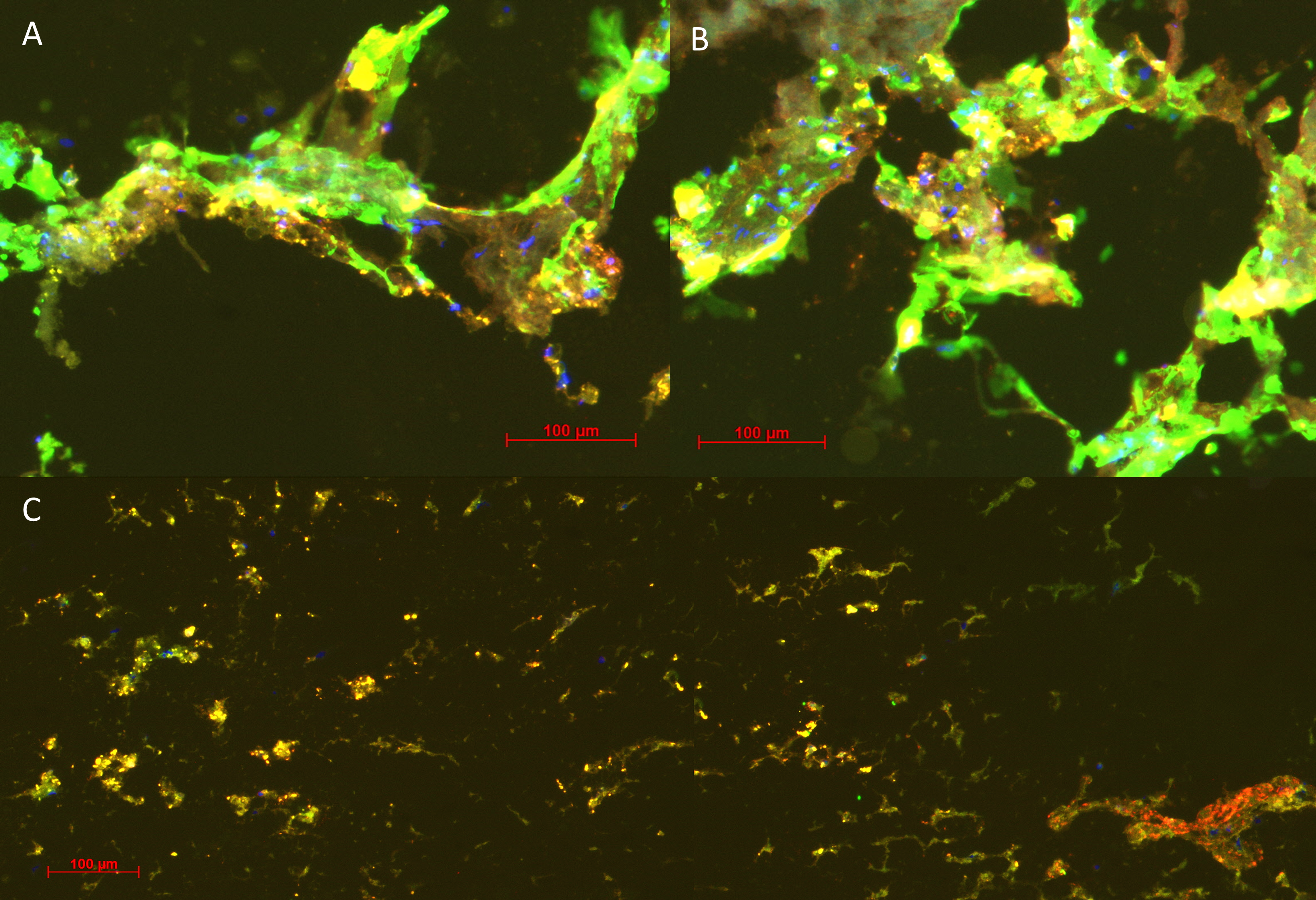

Supplement: Supplementary file 1 — Supplementary file1 (TIF 9654 KB) [file 296_2024_5567_MOESM1_ESM.tif]

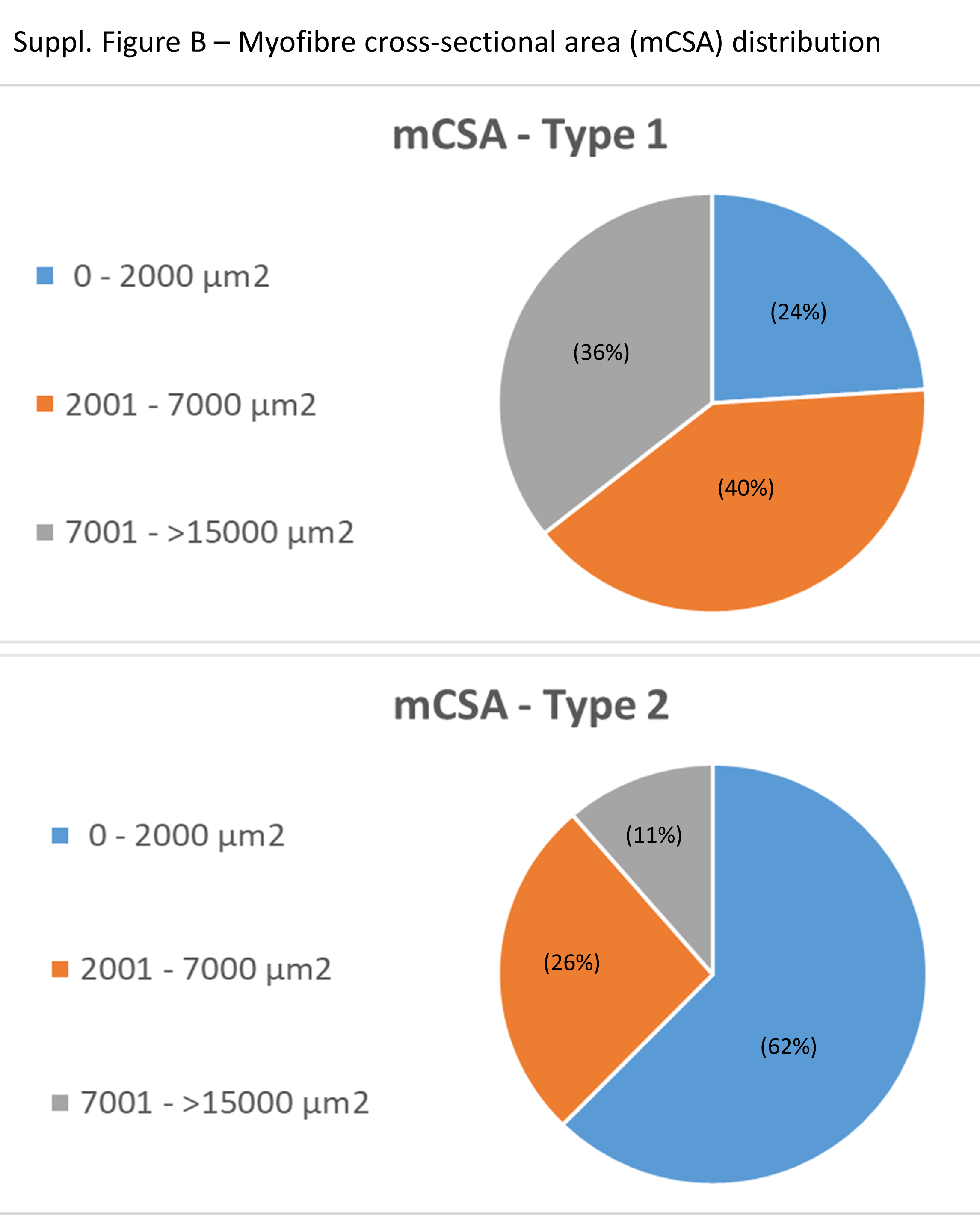

Supplement: Supplementary file 2 — Supplementary file2 (TIF 957 KB) [file 296_2024_5567_MOESM2_ESM.tif]
