# Supplementary material for: Effects of sporadic inclusion body myositis on skeletal muscle fibre type specific morphology and markers of regeneration and inflammation
Source: Rheumatol Int. 2024 Apr 6;44(6):1077–87. doi: 10.1007/s00296-024-05567-8 (PMC11108868; doi:10.1007/s00296-024-05567-8)
Supplement: Supplementary file 3 — Supplementary file3 (DOCX 15 KB) [file 296_2024_5567_MOESM3_ESM.docx]

## Supplementary table A. Antibodies and incubation times.

| Antibody | Origin | Target | Company | Catalogue number | Dilution | Incubation |
| --- | --- | --- | --- | --- | --- | --- |
| Primary |  |  |  |  |  |  |
| Laminin | Rabbit | Basal membrane | DakoCytomation | Z0097 | 1:2000 | Overnight |
| MHC – fast | Mouse | Type II fibres | Sigma-Aldrich | M4276 | 1:1000 | 30 minutes |
| Pax7 | Mouse | Satellite cells | Tokyo institute of technology | 352-523 | 1:200 | Overnight |
| Six1 | Rabbit | Six1^+^ satellite cells | Sigma-Aldrich | HPA001893 | 1:2000 | Overnight |
| Ki67 | Mouse | KI 67^+^ Satellite cells | DakoCytomation | M7240 | 1:100 | Overnight |
| CD68 | Mouse | CD 68+ Macrophages | DakoCytomation | M0814 | 1:400 | Overnight |
| MMR (CD206) | Goat | CD 206+ Macrophages | DakoCytomation | AF2534 | 1:400 | Overnight |
| CD31 | Mouse | Capillaries | DakoCytomation | M0823 | 1:200 | Overnight |
|  |  |  |  |  |  |  |
| Secondary |  |  |  |  |  |  |
| Alexa 555 | Rabbit | Goat primary antibodies | Invitrogen | A21431 | 1:1000 | 60 minutes |
| Alexa 488 | Goat | Rabbit primary antibodies | Invitrogen | A11008 | 1:1000 | 60 minutes |
| Alexa 488 | Donkey | Goat primary antibodies | Invitrogen | A11055 | 1:1000 | 60 minutes |
| Vecta Flour^+^  (amplifier) | Goat | Mouse primary antibodies | Vector | DK-2488 | prediluted | 15 minutes |
| Vecta Flour^+^  (dylight 488) | Horse | Goat antibodies | Vector | DK-2488 | prediluted | 30 minutes |
| Vecta Flour^#^  (amplifier) | Goat | Rabbit primary antibodies | Vector | DK-1488 | prediluted | 15 minutes |
| Vecta Flour^#^  (dylight 488) | Horse | Goat antibodies | Vector | DK-1488 | prediluted | 30 minutes |
| Qdot 625 | Donkey | Rabbit primary antibodies | Invitrogen | Q22086 | 1:2000 | 30 minutes |
| DAPI |  | DNA | Thermo Fischer | 62248 | 1:10000 | 10 seconds |
